# Digital patient-centred learning in medical education: A national learning platform with virtual patients as part of the DigiPaL project

**DOI:** 10.3205/zma001568

**Published:** 2022-09-15

**Authors:** Jacqueline Jennebach, Olaf Ahlers, Angelika Simonsohn, Martin Adler, Julian Özkaya, Tobias Raupach, Martin R. Fischer

**Affiliations:** 1 Medizinischer Fakultätentag der Bundesrepublik Deutschland e.V., Geschäftsstelle, Berlin, Germany; 2 Charité - Universitätsmedizin Berlin, Campus Virchow Klinikum, Klinik für Anästhesiologie m.S. operative Intensivmedizin CCM/CVK, LOOOP-Projekt, Berlin, Germany; 3 LMU-Klinikum, Institut für Didaktik und Ausbildungsforschung in der Medizin, München, Germany; 4 Instruct gGmbH, München, Germany; 5 Universitätsklinikum Bonn (AÖR), Institut für Medizindidaktik, Bonn, Germany

**Keywords:** competency based education, curriculum mapping, virtual patients, problem solving

## Abstract

**Background:** Due to the coronavirus pandemic, the medical faculties in the Federal Republic of Germany converted their curricula to digital formats on a large scale and very quickly in spring 2020 as an emergency measure. At the same time, a start was made on the nationwide exchange of digital teaching/learning materials via the online platform “LOOOP share” in order to save local resources. Among other things, virtual patient cases (VP) were shared across faculties for case-based learning, through which students can acquire clinical decision-making skills.

**Objectives:** Within the framework of the cooperation project “National Learning Platforms for Digital Patient-Related Learning in Medical Studies” (DigiPaL), the usability of VPs for students and teachers should be improved, and the spectrum of disease patterns that are covered by VPs should be systematically expanded.

**Results:** With the participation of many locations, a total of 150 VPs were developed by 96 case authors from 16 faculties, in addition to the existing 403 VPs. The thematic selection was made on the basis of criteria oriented to the National Competence Based Catalogue of Learning Objectives for Undergraduate Medical Education (NKLM). After completion, these VPs were also made available to all faculties for free use via “LOOOP share” and the CASUS learning platform.

**Discussion: **Even after the pandemic, these developed VPs should be available to the faculties and thus make a lasting contribution to improve medical training in Germany – especially in light of digital teaching formats being expressly advocated on the basis of the adapted current Medical Licensure Act (ÄApprO). A possible application is interdisciplinary learning of clinical decision-making with the help of blended learning formats within the framework of a longitudinal curriculum.

The large number of involved colleagues and faculties shows that the nationally coordinated development of VPs across faculties was commonly seen as useful.

## Zusammenfassung

**Hintergrund: **Auf Grund der Coronavirus-Pandemie haben die Medizinischen Fakultäten in der Bundesrepublik Deutschland ihre Curricula im Frühjahr 2020 großflächig und sehr rasch im Sinne eines Notfallplans auf digitale Formate umgestellt. Parallel wurde damit begonnen, digitale Lehr-/Lernangebote über die bereitgestellte Online-Plattform „LOOOP share“ bundesweit auszutauschen, um lokale Ressourcen zu schonen. Unter anderem wurden auch virtuelle Patient*innen-Fälle (VP) für das fallbasierte Lernen fakultätsübergreifend geteilt, durch welche die Studierenden klinische Entscheidungskompetenzen erwerben können.

**Ziele:** Im Rahmen des zu dieser Zeit initiierten Kooperationsprojektes „Nationale Lernplattformen für digitales patientenbezogenes Lernen im Medizinstudium“ (DigiPaL) sollte u.a. die Nutzbarkeit von VP für Studierende und Lehrende verbessert und das Spektrum der verbindlich für die Lehre verfügbaren Krankheitsbilder systematisch erweitert werden.

**Ergebnisse: **Unter Beteiligung vieler Standorte wurden ergänzend zu den bereits bestehenden 403 VP von 96 Fallautor*innen aus 16 Fakultäten insgesamt weitere 150 VP entwickelt. Die thematische Auswahl erfolgte auf Basis von am Nationalen Kompetenzbasierten Lernzielkatalog Medizin 2.0 (NKLM) orientierten Kriterien. Diese VP wurden dann nach Fertigstellung ebenfalls über LOOOP share und die Lernplattform CASUS allen Fakultäten zur freien Nutzung zur Verfügung gestellt.

**Diskussion: **Auch nach der Pandemie sollen diese entwickelten VP den Fakultäten zur Verfügung stehen und so nachhaltig zur Verbesserung der medizinischen Ausbildung in der Bundesrepublik Deutschland beitragen – insbesondere vor dem Hintergrund, dass auf Basis der angepassten aktuellen Ärztlichen Approbationsordnung (ÄApprO) digitale Lehrformate ausdrücklich befürwortet werden. Denkbar ist perspektivisch das gezielte interdisziplinäre Erlernen klinischer Entscheidungsfindung mithilfe von Blended Learning Formaten im Rahmen eines longitudinalen Curriculums.

Die große Zahl beteiligter Kolleg*innen und Fakultäten zeigt, dass die fakultätsübergreifende, national abgestimmte Entwicklung von VP als sinnvoll angesehen wurde.

## 1. Introduction

In the course of the restrictions imposed by the coronavirus pandemic, the “Act on the Protection of the Population in the Event of an Epidemic Situation of National Importance” was passed, which was intended to ensure the training of doctors, among other things [http://www.bgbl.de/xaver/bgbl/start.xav?startbk=Bundesanzeiger_BGBl&jumpTo=bgbl120s0587.pdf]. Based on this, §2 of the “Deviations from the Medical Licensing Act” explicitly created the legal option to use digital teaching formats as a substitute for theoretical face-to-face teaching formats and as a supplement for practical teaching formats in human medicine degree programmes [https://www.gesetze-im-internet.de/epi_appro2002abwv/index.html]. However, the use of this option posed great challenges for the medical faculties in the spring of 2020:

1. many digital learning materials (“resources”) were needed at each faculty at short notice,
2. the use of these resources had to be aligned with the content of their own curriculum; and
3. in particular, the core competence “Clinical Decision-Making” should be acquirable digitally if possible.

All three of the above challenges were addressed by the team of authors in a two-step process. In a first step, the faculties were supported in the short term in the implementation of teaching during the pandemic. This step was the basis for step 2 presented in this project report. Step 2 ensures the sustainability of the project – especially with regard to the requirements of the new Medical Licensure Act (ÄApprO), whose entry into force is currently planned for 2025.

### 1.1. Step 1: Short-term nationwide provision of already existing online resources

The aim of this first step was to make already existing online resources available for use by faculties nationwide as quickly as possible. This should be possible free of charge via standard browsers as well as mobile devices. In addition, the faculties should be enabled to easily compare the resources offered with their own curricula in order to be able to identify suitable supplements to their own face-to-face courses. One focus was the integration of virtual patient cases (VP) with regard to learning clinical decision-making as an important medical competence.

#### 1.1.1. Networking of existing online resources (addressing challenge (1))

In March 2020, the internationally used LOOOP online platform was adapted by the team of developers at Charité – Universitätsmedizin Berlin so that digital resources can be shared and used by all faculties nationwide (see [https://looop-share.charite.de]). For this purpose, the faculties can upload links to their online accessible digital resources on “LOOOP share” and control the access rights to these resources. The platform was adapted in close consultation with the Association of Medical Faculties in Germany (MFT).

Since June 2020, access to “LOOOP share” has been possible via the authentication and authorisation infrastructure of the German Research Network (DFN-AAI, see [https://doku.tid.dfn.de/de:aai:about]) on a uniform nationwide basis within the framework of a “Single Sign On”. This low-threshold control of access rights was made possible by a grant from the Federal Ministry of Health (BMG), which has been in place since 01 June 2020, as part of the cooperation project “National Learning Platforms for Digital Patient-Related Learning in Medical Studies” (DigiPaL).

##### 1.1.2. Use of online resources for own curriculum (addressing challenge (2))

To enable content-based navigation through the online resources linked in “LOOOP share”, each resource was individually mapped to the content of the National Competence Based Catalogue of Learning Objectives for Undergraduate Medical Education (NKLM, version 1.0) [1] published in 2015. This mapping was carried out according to a concept for curricular mapping that has been developed since 2004 under the leadership of the Charité – Universitätsmedizin Berlin within the framework of the international LOOOP research network [2]. The basis of this concept, which is currently being used to map approx. 140 degree programs in 22 countries (see [https://looop.charite.de]), includes preliminary work by Harden [3] and Willett [4]. 

Faculties that have already mapped their own curricula against the NKLM 1.0 as part of the LOOOP research network will automatically be shown the resources that match their courses (linked to the same NKLM content). Faculties that do not map using LOOOP can identify suitable resources in “LOOOP share” via the navigation within the thematically structured NKLM.

##### 1.1.3. Virtual patient cases (addressing challenge (3))

VPs are an effective way to learn, train and test clinical reasoning and, in particular, clinical decision-making [5], [6], [7]. The CASUS learning platform has been used for case-based learning using VPs since 1993 [8] and has been continuously developed (see [https://www.instruct.eu/#casus_software]). For example, special functions for clinical decision-making were added as part of a separate EU-funded project (see [https://www.foliospaces.org/view/view.php?id=56393]). In the CASUS learning environment, a comprehensive pool of VPs was available in spring 2020, which had been created by six medical faculties.

As part of step 1 at the beginning of the pandemic, the faculties deposited 403 completed VPs mapped against the NKLM 1.0 on “LOOOP share”, for which cross-site use was possible under consideration of legal framework conditions (e.g. copyright) and for which case creators saw no need for revision. These VPs addressed 94 diseases of the NKLM - primarily addressing internal medicine, surgery and paediatrics. Since May 2020, those VPs have been accessible nationwide free of charge via DFN-AAI in CASUS. Table 1 (Tab. 1) shows the increasing number of accesses to these VPs during the period between the summer semester 2020 and the summer semester 2021. 

#### 1.2. Step 2: Content coverage for patient-related teaching by means of VPs and assurance of medium- and long-term usability 

The amendments to the currently valid ÄApprO [http://www.bgbl.de/xaver/bgbl/start.xav?startbk=Bundesanzeiger_BGBl&jumpTo=bgbl121s4335.pdf] published in September 2021 and the drafts for the new ÄApprO, which is planned to come into force in 2025, explicitly allow the use of digital teaching formats and thus enable sustainable use of the innovations described in step 1 even beyond the pandemic. The DigiPaL project is intended to support the faculties in using this possibility. 

To this end, the number of already existing VPs should be increased in order to align the content of the VP pool, which can be used nationwide, specifically with the requirements of the new ÄApprO.

##### 1.2.1. Content requirements of the new Medical Licensure Act and NKLM 2.0

When the new ÄApprO comes into force, the content of the curricula at the medical faculties is to be based approx. 80% on the then available version of the NKLM (so-called "core curricula"). Therefore, between 2018 and 2021, the NKLM 1.0 was further developed into the interim version NKLM 2.0 in a nationwide process involving many hundreds of experts from medical faculties and professional societies and published in April 2021 [https://www.nklm.de]. This further development of the NKLM took place within the framework of a standardised process [https://nklm.de/zend/objective/list/orderBy/@objectivePosition/modul/200557] together with the development of the Competence-Oriented Subject Catalogue and was coordinated by the NKLM office (GSt.) at the MFT.

Due to the complexity of the NKLM, only the relevant NKLM sections and detailed information are described below: 

The NKLM 2.0 defines a selection of diseases relevant for the core curricula of the faculties. These are in turn considered on the basis of individual clinical aspects (so-called “descriptors”), such as diagnostics, therapy, emergency treatment and management. Not all descriptors were marked as “relevant” for each disease in the NKLM. This is mainly due to two reasons: Firstly, certain aspects simply do not exist for some diseases, e.g. not every disease requires an emergency treatment. Secondly, only those aspects were marked as “relevant” in the NKLM which should already be learned by medical students. Additionally, a decision was made for each descriptor classified as “relevant” as to whether a cognitive (for example, the preparation of a patient-related diagnostic or therapy plan) or practical action competence should be acquired. Furthermore, for each disease it was marked whether it was a rare disease.

Cross-links from each individual disease to other chapters of the NKLM were then used to define which diagnostics, therapy, etc. should be learned in the context of this disease. The diseases listed in the NKLM thus play a central role both in defining the clinical content of medical studies and in selecting the aspects of clinical decision-making relevant to the degree programme.

##### 1.2.2. Project goals

As part of the BMG-funded DigiPaL project, the VP pool available nationwide via LOOOP share in 2020 should be expanded in such a way that, after expansion, it covers the diseases with relevant clinical action competencies in NKLM 2.0. The decision as to which VPs are additionally required should be made on the basis of standardised criteria (see 2.2) using an analysis of the VPs already available in “LOOOP share”. In order to identify the corresponding diseases, standardised criteria should be developed with regard to practical action competencies. Subsequently, all medical faculties nationwide should be able to participate in this process.

## 2. Project description

### 2.1. Establishing the decision-making and organisational structures

After approval of the DigiPaL project application by the BMG, which in addition to the project described here also included the development of a digital virtual emergency department (DIVANA), an “editorial board” was set up. This board was to approve the NKLM-based criteria to be developed for the purpose of selecting the disease topics for the VPs and also decide on the subsequent case allocation to the faculties. The case allocation was carried out by the MFT, the coordination of the VP revision or new development was taken over by the Institute of Medical Education at LMU Munich.

#### 2.2. Development of standardised criteria for VP revision/creation

Standardised criteria for the selection of the most important diseases (and their descriptors) to be covered by VPs were developed by the GSt. on the basis of the NKLM 2.0. If one or more of the criteria shown in table 2 (Tab. 2) were met, a disease was included in the selection. The basic requirement was that it was not marked as a rare disease. 

In the criteria, emphasis was generally placed on marking an action competence in order to take account of the VP’s goal of acquiring competence in the area of “clinical decision-making”. 

The combination of the descriptors “diagnostics” and “therapy” in criterion 1 was due to the fact that a clinical process can be represented very well, if action competence is required with regard to both descriptors. The same applies to the combination of the descriptors “diagnostics” and “management” in criterion 3.

The descriptor “emergency therapy” includes both emergency diagnostics (or first of all the recognition of an emergency situation) and the initiation of first therapeutic steps – which is why this descriptor was considered in criterion 2. Here, however, the other descriptors were not relevant in combination, as there are many diseases for which it is only important for students to recognise the acute situation and initiate measures – but not to be able to carry out the general diagnostics and therapy of these sometimes complex diseases by themselves.

#### 2.3. Analysis of required VPs and comparison with the already published VP pool

Using the criteria presented in 2.2, those diseases and descriptors of the NKLM 2.0 were filtered out that should be covered by VPs. With the help of the successor relationships to NKLM 1.0 defined in NKLM 2.0, the diseases of both catalogue versions were then compared with each other and the respective coverage by VPs already published in “LOOOP share” was analysed. The “gaps” identified in this coverage analysis could refer both to diseases not yet covered by VPs and to descriptors of already existing diseases not yet covered in published VPs.

Based on these analyses, an existing pool of previously unpublished or no longer used older VPs (with extensive need for revision) was first searched for 75 suitable cases to cover the necessary topics. Subsequently, 75 new VPs were defined to be created in order to close the then remaining “gaps”. 

In addition, a standardised procedure was adopted by the Editorial Board to determine how to proceed before applying the above criteria if – as is to be expected – not exactly 75 VPs needing revision and 75 new VPs are identified.

1. If too few VPs are identified from the analyses, “multifaceted diseases” should be added, even if they are already addressed in an existing CASUS case. These VPs should then cover an alternative disease focus.
2. If too many VPs were identified, the Editorial Board could exclude VPs that a) were less suitable for implementation as VPs from a content perspective or b) were already covered by enough similar VPs.

#### 2.4. Tendering of the VPs and assignment to faculties

The 11 case packages (corresponding to the organ system chapters of the NKLM 2.0) were then tendered nationwide and distributed by the Editorial Board to preferably two applying faculties. These faculties were each to work on half of the advertised VPs and be available to each other as review partners. 

The faculties received an allowance of 1,200 € for each newly created VP and 600 € for each VP needing revision. The reciprocal case review was included in this amount.

#### 2.5. Creation of the VPs

The case authors received comprehensive training from the LMU, which included both the didactic design of VPs and the use of the CASUS authoring system. The 96 authors were individually accompanied throughout the entire process of case development, and all VPs received a formative didactic review with detailed revision suggestions after their first completion.

#### 2.6. Provision of the VPs nationwide

After finalising the respective VPs, the authors were asked to provide short summaries for “LOOOP share”. In addition, a mapping of the new/revised VPs against the NKLM 1.0, supported by the GSt, took place so that these VPs can also be easily integrated into the curriculum by the faculties on the basis of the summaries and the previous mapping. 

## 3. Results

### 3.1. VPs needing revision and new VPs to be created

#### 3.1.1. Selection of the VPs needing revision

From the pool of VPs needing revision, 50 suitable cases were identified with the help of the criteria described under 2. The number of suitable VPs per disease to be covered was between 1 and 4 (see table 3 (Tab. 3)) and a maximum of 2 VPs per disease were selected (finally 44 VPs needing revision were identified). Subsequently, further VPs needing revision were defined according to 2.3.1. and thus, the target of 75 VPs needing revision was reached. 

##### 3.1.2. Definition of the new VPs to be created

Within the framework of the procedure described under 2., a total of 81 diseases were identified that were neither covered by VPs already published nor by VPs needing revision. As described in 2.3.2, these diseases were then reduced to 75. Table 4 (Tab. 4) presents the six diseases (incl. the respective justification) for which no VPs were created.

#### 3.2. Distribution of the VP revision and redrafting among the faculties

A total of 16 faculties applied in the tender process and were assigned to the 11 organ-related thematic packages (see table 5 (Tab. 5)). Table 6 (Tab. 6) provides an overview of the thematic allocation of the faculties.

#### 3.3. Created VPs

All 150 VPs needing revision and new VPs were created between 01.11.2020 and 12.12.2021, peer reviewed and linked in “LOOOP share”.

## 4. Discussion

The pool of VPs available free of charge nationwide was expanded within the framework of the DigiPaL project so that the most important aspects of the diseases listed in the NKLM are now covered by VPs. These VPs are accessible via the DFN-AAI with the help of the “LOOOP share”-platform, which is also available free of charge, and can now be used for decentralised and supplementary teaching by the faculties. 

The format of the VP is particularly suitable for training of clinical decision-making (regarding diagnosis and therapy decisions). Thematically self-contained case collections represent a particular added value. The case collection on occupational and environmental medicine, which has been used at many faculties since 2007, is a successful example of this cross-faculty use of VPs [9]. However, VPs can generally only be used to illustrate cognitive action competence (i.e., for example, the independent creation of diagnostic and/or therapy plans) [10]. Therefore, supplementary teaching of procedural and manual skills (handling; practice through simulations) is necessary to enable successful implementation of what has been learned [10], [11].

Mapping the VPs to the NKLM made sense considering the NKLM is expected to become the binding basis for the nationwide core curricula after a further revision in 2025. The conception of the criteria was based on the assumption that initially only those diseases should be considered that students regularly encounter. The VP situation was intended as a “complaint-related consultation” and not in the sense of a preventive medical presentation. Furthermore, special importance was attached to a required “competence to act”, since a certain independent action is expected of the students at the end of their studies.

The criteria were developed and applied on the basis of the interim status of the NKLM at the end of 2020. It would be possible to compare whether the VPs to be included would have changed after finalisation of the NKLM 2.0 by April 2021. In view of the further development of the NKLM planned until 2025, however, it seems reasonable to carry out a review of the VPs selected by the developed criteria on the basis of a later version of the NKLM (e.g. version 2.1 planned for 2024) in order to adjust the pool of VPs according to the core curricular content. In addition, the development of VPs on the basis of the other NKLM diseases (which do not fit the criteria mentioned) is of course also possible in order to give students the opportunity to deal with these topics.

One limitation of the DigiPaL project could be the focus of the VsP on diseases (instead of, for example, on consultation occasions in the sense of “leading symptoms”). This approach was due to the time pressure in the course of the pandemic, in order to enable rapid usability of the VPs. A focus on consultation causes would have meant that the lecturers would have had to look through all the published VPs with a suitable symptom to see whether they were actually related to the diagnosis they were to teach. In the context of the “re-mapping” of the VPs against the NKLM version 2.1 already considered above, however, an additional marking of the consultation occasions addressed in a VP would make sense.

## 5. Conclusion

The nationwide participation of 17 faculties reflects the great interest in the format of the VP as a component of a canon of methods for teaching clinical decision-making skills, which was certainly increased in the course of the special demands during the coronavirus pandemic. Considering the low expense allowance for the participating faculties, a certain intrinsic motivation of the participants can be assumed. Additionally, a certain amount of faculty and specialization visibility may also be a motivating factor. In our view, this shows that the expected resource-saving synergy effects of cross-faculty planning and use of VPs have been accepted by many faculties. The rising usage of VPs also implies that the anticipated importance of VPs (especially during the decentralised teaching situation) was correctly assessed. 

Since all relevant aspects of the diseases defined in the project are now covered by VPs, the faculties already have a VP pool that can be used across the board as a methodological supplement to the established patient-related teaching formats (e.g. in the context of blended learning). This can also be used specifically for learning clinical decision-making after returning to face-to-face teaching, and can be easily adapted to each new version of the NKLM with the help of the developed criteria.

As part of the DigiPaL project, the foundation was thus laid for a significant expansion of digital teaching within the framework of the new ÄApprO on the basis of the NKLM.

## Authors contributions

Jacqueline Jennebach and Olaf Ahlers contributed equally.

## Acknowledgements

The authors would like to thank – also on behalf of the students and lecturers at the medical faculties – all colleagues who have made this extensive project possible through their commitment.

## Funding

DigiPaL was supported by the German Ministry of Health on the basis of a resolution of the German Bundestag under the funding code 2520COR200.

## Competing interests

The authors declare that they have no competing interests. 

## Figures and Tables

**Table 1:**
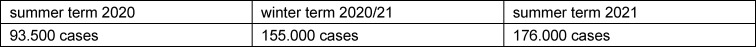
Number of virtual patient case usages in CASUS

**Table 2:**
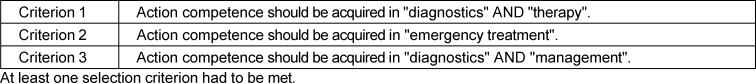
Standardised selection criteria

**Table 3:**
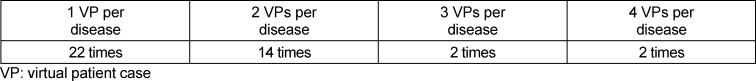
Number of available virtual patient cases per disease

**Table 4:**
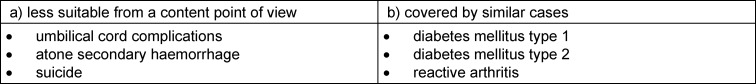
Diseases for which no virtual patient cases were created

**Table 5:**
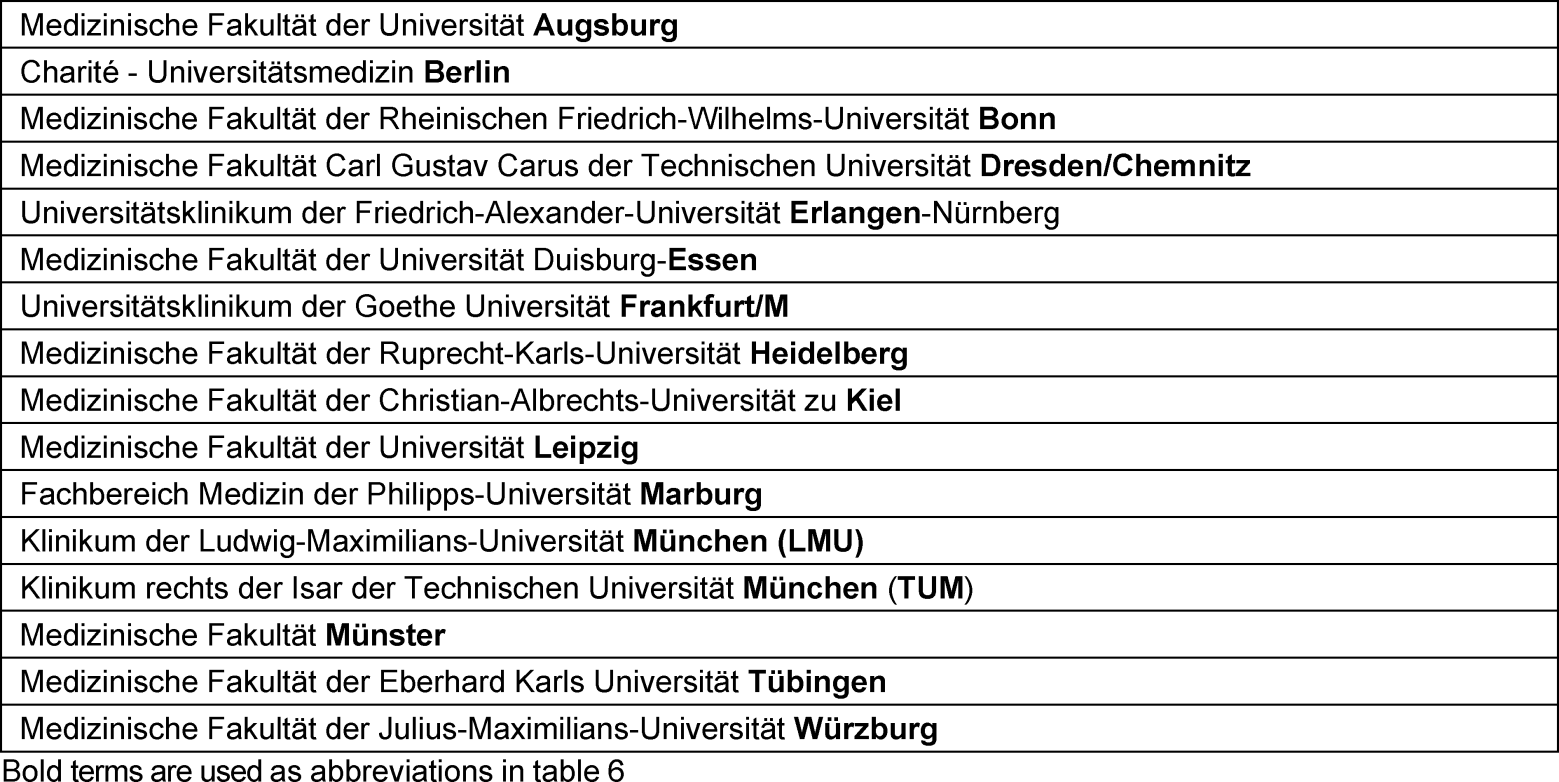
Overview of the participating faculties

**Table 6:**
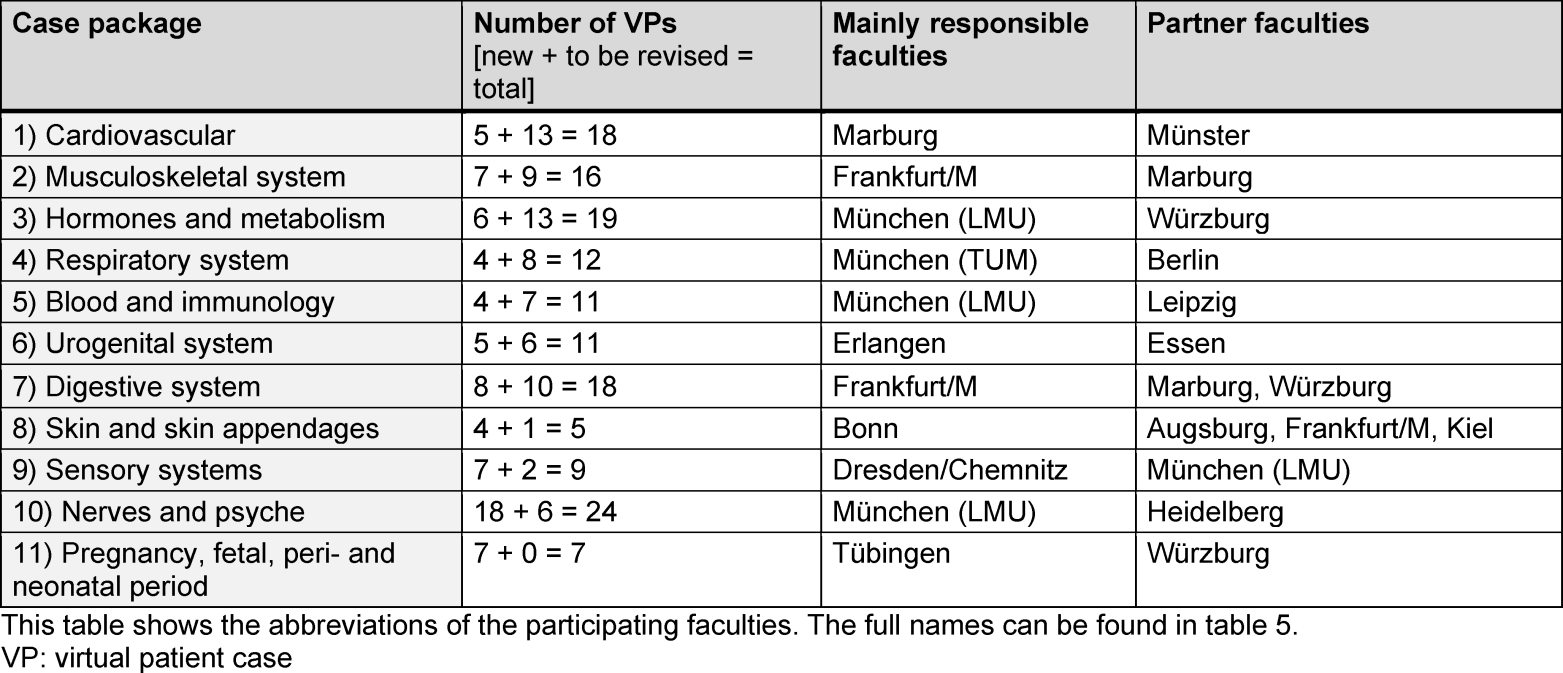
Overview of the virtual patient cases covered by the faculties
